# Translation, cross-cultural adaptation and psychometric validation of the Thai version of the STarT Back Screening Tool in patients with non-specific low back pain

**DOI:** 10.1186/s12891-021-04347-w

**Published:** 2021-05-18

**Authors:** Taweewat Wiangkham, Nattawan Phungwattanakul, Natthathida Thongbai, Nisa Situy, Titipa Polchaika, Isara Kongmee, Duangporn Thongnoi, Rujirat Chaisang, Wanisara Suwanmongkhon

**Affiliations:** 1 Exercise and Rehabilitation Sciences Research Unit, Faculty of Allied Health Sciences, Naresuan University, Phitsanulok, 65000 Thailand; 2 Department of Physical Therapy, Faculty of Allied Health Sciences, Naresuan University, Phitsanulok, 65000 Thailand; 3 Department of English Language, Faculty of Humanities, Naresuan University, Phitsanulok, 65000 Thailand; 4 Department of Accounting, Faculty of Business Administration, Chiang Mai University, Chiang Mai, 50200 Thailand

**Keywords:** Low back pain, Cross-cultural adaptation, Psychometric properties, STarT Back screening tool-Thai version, SBST-TH

## Abstract

**Background:**

Low back pain (LBP) is a top musculoskeletal problem and a substantial cause of socioeconomic burden internationally. The STarT Back Screening Tool (SBST) is a useful screening tool to manage patients with LBP but it is unavailable in Thai. Therefore, the aims of this study were to translate and cross-culturally adapt the SBST into a Thai version (SBST-TH) and validate its psychometric properties (e.g., factor analysis, internal consistency, test-retest reliability, agreement, convergent validity and discriminative validity).

**Methods:**

Translation and cross-cultural adaptation of the SBST into Thai version were conducted according to standard guidelines. A total of 200 participants with non-specific LBP were invited to complete the SBST, visual analogue scale for pain intensity, Roland-Morris disability questionnaire (RMDQ), fear-avoidance beliefs questionnaire, pain catastrophising scale, hospital anxiety and depression scale and the EuroQol five-dimensional questionnaire. Thirty participants completed the SBST-TH twice with an interval of 48 h to evaluate test-retest reliability.

**Results:**

Factor analysis demonstrated two (physical and psychological) components for the SBST-TH (39.38% of the total variance). The Cronbach’s alpha (0.86 for total score and 0.76 for psychosocial subscore) represent satisfactory internal consistency. The acceptability of intraclass correlation coefficient was found in the total (0.73) and subscore (0.79). The areas under the curve (AUC) for the total score ranged 0.67–0.85 and 0.66–0.75 for subscore. The excellent discriminative validity was observed (AUC = 0.85, 95% confidence interval = 0.72, 0.97) between the total score of the SBST-TH and disability (RMDQ). Spearman’s correlation coefficients represented moderate to strong correlation (0.32–0.56) between the SBST-TH and all questionnaires. The findings suggest a good relationship between the SBST-TH and disability and quality of life. Owing to the results from the convergent and discriminative validity, construct validity of the SBST-TH can be supported. The minimal detectable changes of the total score and subscore were 2.04 and 1.60, respectively. Significant floor and ceiling effects were not found in the SBST-TH.

**Conclusion:**

The SBST-TH was successfully translated and adapted. It is a valid and reliable tool to classify Thai patients with non-specific LBP into low, moderate and high risks for chronicity.

**Trial registration:**

TCTR20191009005#.

## Background

Low back pain (LBP) is a top musculoskeletal problem internationally [1] and a substantial cause for disability [2], especially non-specific LBP [3]. Individuals with non-specific LBP can experience pain, functional limitation and reduced quality of life (QoL). This can lead to socioeconomic burden internationally. In the US, approximately $70 billion of the indirect cost is spent in managing LBP. Around £4.1 and £3.1 billion are the costs of LBP management in Switzerland [4] and the Netherlands [5], respectively. Interestingly, the current evidence reports the burden of LBP, recurrence of symptom(s) and chronicity worldwide [1, 2]. In Thailand, musculoskeletal disorders are the fourth major health problem, following cardiovascular disease, diabetes, and chronic respiratory disease. Remarkably, LBP is reported as the second most common musculoskeletal problem, following soft tissue disorders [6].

Individuals with non-specific LBP can suffer from physical (e.g., pain and disability) and psychological problems (e.g., pain catastrophising, anxiety, depression and fear-avoidance beliefs) [7]. Consequentially, both components should be addressed to manage individuals with non-specific LBP. In primary care, treatment focused on biopsychosocial problems is an effective management among patients with LBP [8–10]. Thus, assessments in physical and psychological perspectives should be addressed in patients with non-specific LBP.

The Keele STarT Back Screening Tool (SBST) is a simple prognostic screening tool evaluating both physical and psychosocial aspects for LBP patients [11]. It is developed to classify patients with LBP into low, moderate and high risks according to risk for chronicity to help primary care management in the UK [10]. The findings demonstrated that the SBST can be used to reduce disability and cost of management in patients with LBP, approximately £675 per one patient for socioeconomics burden and approximately £34.39 for the health services [10]. It can be seen that the SBST can induce appropriate treatment, save time, and decrease the costs of LBP management owing to the stratified care [10].

The SBST is a self-assessment tool with nine items (items 1–4 assessed the physical factors and items 5–9 assessed the psychosocial factors) [10]. The total score ranges from 0 to 9 [10]. Patients with a total score ≤ 3 will be classified into a low-risk group, whereas patients with a total score ≥ 4 and a psychosocial subscore ≤3 will be classified into a medium-risk group [10]. A total score ≥ 4 and a psychosocial subscore ≥4 will be classified into a high-risk group [10]. An appropriate treatment will be designed for patients in each risk group: low-risk group (e.g., self-management and providing information for education), medium-risk group (e.g., physiotherapy to manage pain, functional impairment and disability) and high-risk group (e.g., physiotherapy to manage pain, functional impairment, disability and distress) [10]. Although the SBST has been translated into 38 languages, a Thai version of the SBST is not available to help the primary care management of Thai patients with LBP. Therefore, the aims of this study were to translate and adapt the original version of the SBST into a Thai version and test its psychometric properties in patients with non-specific LBP.

## Methods

This study was divided into two stages: 1) linguistic translation and cross-cultural adaptation of the STarT for Thai patients with LBP and 2) tests of its psychometric properties. All processes were performed in accordance with the Declaration of Helsinki. The permission for translation and cross-cultural adaptation of the STarT was obtained from the Arthritis Research UK Primary Care Centre, United Kingdom, via e-mail. See the following link for further details: https://startback.hfac.keele.ac.uk/training/resources/start-back-translations/.

### Stage I: linguistic translation and cross-cultural adaptation

The translation and cross-cultural adaptation were conducted based on the standard guidelines [12]. Forward translation of the original English version of the STarT into Thai was independently performed by two bilingual translators (Thai and English but Thai first language). The first translator was a musculoskeletal physiotherapist (PhD physiotherapy qualification with 12 years of experience) who is familiar with the STarT (provided T1 version). The second translator was an English lecturer (PhD linguistic qualification) and professional translator (provided T2 version). Both translators and the three researchers synthesised the T12 version with a written report prior to back translation. Two back translators (PhD linguistic qualification) who were bilingual in Thai and English with no medical background independently translated the T12 version to English without knowledge of the original English version. In the next step, study committee (consisted of the four translators, three researchers, one musculoskeletal physiotherapist and one expert linguistic chair) discussed the original questionnaire, all translational versions and written reports until agreement on the semantic, idiomatic, experiential and conceptual equivalences between the original and targeted versions in order to establish a pre-final version. The pre-final version was tested to assess the clarity of the instruction, comprehensiveness of the items, cultural appropriateness and acceptability using a qualitative cognitive debriefing on 30 patients with LBP who were not included in the data analysis. Comprehension difficulties and further modifications were not reported to the research team. Thus, the Thai version of the SBST was ready for psychometric testing.

### Stage II: psychometric properties of the final version

#### Participants

Potential participants aged 20–70 years old with LBP were recruited from July to December 2019. Individuals with a history of spinal surgery, spinal myelopathy, vertebral fracture, tumours, systemic diseases with possible effect on the musculoskeletal system, pregnancy, and clinically cognitive impairment or who were unable to complete the questionnaire independently were excluded. The participant information sheet and consent form were given to potential participants to provide an opportunity to ask questions, confirm eligibility and obtain written consent. Participants provided written informed consent prior to participation.

### Instruments

Participants were evaluated using the Thai version of all questionnaires.

#### STarT Back screening tool (SBST)

The SBST is a fast, simple, valid and reliable screening tool with nine brief items in terms of referred leg pain, comorbid pain, difficulties in walking, difficulties in dressing, fear of physical activity, anxiety, pain catastrophising, depressive mood and overall impact of pain [11]. It was developed to stratify patients into matched treatment pathways based on their prognosis [10]. The SBST response options are dichotomous (agree/disagree) except the ninth item, which is a five-point Likert scale. Prognostic factors covering both physical and psychological aspects are addressed in items 1–4 and items 5–9, respectively. Patients can be classified into three groups (low, medium and high risk for chronicity). Those allocated to the high-risk subgroup are at greater risk of persistent disabling LBP symptoms. The total score ranges from 0 to 9 and less than four points is classified as low risk. Patients will be classified as medium or high risk if the total score is higher than three. If the psychological subscore (items 5–9, range from 0 to 5) is less than four, the patients will be classified as medium risk; if the sum is equal to or greater than four, patients will be classified as high risk [11].

#### Visual analogue scale (VAS) for pain intensity

VAS is a simple and common method with validity and reliability (ICC = 0.97) to assess pain intensity [13, 14]. It is a 10-cm horizontal line with “no pain” written at the left end point and “worst imaginable pain” at the right end point. Participants were asked to draw a vertical line to mark a point corresponding to the magnitude of their current pain.

#### Roland-Morris disability questionnaire (RMDQ)

The RMDQ is a common self-report questionnaire to evaluate LBP disability with 24 (yes/no) items [15]. The total score ranges from 0 to 24, with a higher score indicating greater disability. The questionnaire was translated and tested for reliability (*n* = 120, Cronbach’s alpha = 0.83, range 0.71–0.93) by Jirarattanaphochai et al. for Thai patients with LBP [16].

#### Fear-avoidance beliefs questionnaire (FABQ)

The FABQ is a self-report questionnaire with 16 items (each scored 0 to 6) covering both work and physical activity [17]. It is a valid and reliable (Cronbach’s alpha = 0.88 for work and 0.77 for physical activity, ICC for test-reliability = 0.74) tool to assess disability in patients with LBP [17]. The FABQ was translated and cross-culturally adapted into a Thai version and was reported as a valid and reliable tool (*n* = 129, Cronbach’s alpha range 0.87–0.88, ICC_2,1_ = 0.986) [18].

#### Pain Catastrophising scale (PCS)

The PCS is a valid and reliable self-report tool (Cronbach’s alpha = 0.87) with 13 items focusing on thoughts and feelings to measure catastrophising [19]. It contains a three-factor solution: rumination (4 items, Cronbach’s alpha = 0.87), magnification (3 items, Cronbach’s alpha = 0.60) and helplessness (6 items, Cronbach’s alpha = 0.79). Each item comprises a five-point Likert scale, ranging from 0 (not at all) to 4 (all the time), resulting in 0–52 points for a total score. A higher score indicates higher pain catastrophising. The PCS was adapted into Thai with good validity and reliability (Cronbach’s alpha = 0.93, 0.84, 0.74 and 0.85 for total score, rumination, magnification and helplessness subscales, respectively) [20].

#### Hospital anxiety and depression scale (HADS)

The HADS is a valid and reliable tool to assess anxiety (7 items, Cronbach’s alpha = 0.83) and depression (7 items, Cronbach’s alpha = 0.82), which is an important prognostic factor for LBP [21, 22]. The total score ranges from 0 to 42, with individual scores ranging from 0 to 3 (a four-point response scale). Higher scores indicate higher levels of anxiety and depression. The HADS was translated and adapted into Thai, demonstrating a valid and reliable tool (Cronbach’s alpha = 0.86 for anxiety and 0.83 for depression) to assess anxiety and depression among Thai patients [23].

#### EuroQol five-dimensional five-level questionnaire (EQ-5D-5L)

The EQ-5D-5L is a valid and reliable self-report QoL questionnaire [24–26]. It is recommended as a useful tool for measuring general health status with five dimensions in terms of mobility, self-care, usual activities, pain/discomfort and anxiety/depression. The score ranges from 0 (dead) to 1 (perfect health). The EQ-5D-5L has been translated into many languages, including Thai and it is a valid and reliable tool (ICC_2,1_ = 0.70) [27, 28].

### Statistical analysis

Descriptive statistics (percentage, means and standard deviation) were used to illustrate participants’ demographic characteristics. SPSS statistical package (version 17) was used to analyse validity and reliability as the following.

### Content validity

Content validity was evaluated by the expert committee panel in the translational stage. Floor and ceiling effects (> 15% of respondents who achieved the lowest or highest possible SBST-Thai version (SBST-TH) total score were considered) and missing data were identified to evaluate the acceptability [29].

### Factor analysis

Exploratory factor analysis was performed to explore the dimensionality of the SBST-TH using principle component analysis with varimax rotation method. Eigenvalue ≥1 and items of loading ≥0.4 were considered for satisfactory factors, which were named based on included items and their factor loading. Statistical significance (*p* <  0.05) of Bartlett’s test of sphericity represents appropriate factor analysis. The Kaiser-Meyer-Olkin (KMO) ranges from 0 to 1, with higher than 0.5 indicating good factor analysis [30].

### Internal consistency

Internal consistency of the SBST-TH was evaluated using Cronbach’s α coefficient. A Cronbach’s α value higher than 0.7 was acceptable [29].

### Test-retest reliability and agreement

Test-retest reliability of the SBST-TH was evaluated using intraclass correlation coefficient (ICC)_2,1_ in 30 participants. The participants were invited to complete the questionnaire twice with an interval of 48 h to minimise any memory of previous answer and variations in clinical conditions [18]. ICC can range from 0 to 1. The following criteria were used to interpret the ICC: 0: no reliability, < 0.5: unacceptable, 0.5 - < 0.6: poor, 0.6 - < 0.7: questionable, 0.7 - < 0.8: acceptable, 0.8 - < 0.9: good, ≥0.9: excellent and 1: perfect reliability [31]. The standard error of measurement (SEM = standard deviation of all test scores × $\sqrt{1- ICC}$) and minimal detectable change (MDC= $1.96\times \sqrt{2}\times SEM$) were calculated to estimate the absolute reliability [32].

### Convergent validity

The correlations between the SBST-TH and VAS (pain intensity), RMDQ, FABQ, PCS, HADS and EQ-5D-5L were examined using Spearman rank correlation coefficient (*rho*). The correlations were defined as strong (*rho* ≥ 0.5), moderate (0.3 *≤ rho* <  0.5) or weak (*rho* <  0.3) [33]. According to the previous versions of the SBST [8, 34–37], the scores of the SBST-TH were expected to be significantly correlated to all questionnaires.

### Discriminative validity

Areas under the curve (AUC) were calculated to evaluate discriminative validity between the SBST-TH and the RMDQ, FABQ, PCS and HADS. The cut-offs were defined as follows: RMDQ ≥7 [11], FABQ ≥43 [38], PCS ≥ 20 [39], and HADS ≥8 [8]. An AUC = 0.5 indicated “no discrimination”, 0.7 to < 0.8 was considered “acceptable discrimination”, 0.8 to < 0.9 was considered “excellent discrimination” and ≥ 0.9 indicated “outstanding discrimination” [40]. According to the previous versions of the SBST [8, 41–43], the scores of the SBST-TH were expected to be discriminated to the RMDQ, HADS and PCS.

## Results

A total of 200 patients with non-specific LBP (42 males, 158 females) participated in this study. The demographic and clinical characteristics of the participants are presented in Table 1.

**Table 1:** Demographic and clinical characteristics of the participants (*n* = 200)

Variables	% (n)	Mean ± SD	Minimum	Maximum
Age (year)		24.95 ± 8.75	20.00	62.00
Genders				
Female	79.00 (158)			
Male	21.00 (42)			
Education				
No primary school	–			
Primary school	1.50 (3)			
High school	91.00 (182)			
Bachelor’s degree	6.00 (12)			
Master’s degree	1.50 (3)			
Doctoral degree	–			
Types of low back pain				
Acute	52.00 (104)			
Sub-acute	16.00 (32)			
Chronic	32.00 (64)			
STarT risk group				
Low risk	80.00 (160)			
Medium risk	16.00 (32)			
High risk	4 .00 (8)			
VAS (0–100)		3.11 ± 1.57	0.40	7.40
SBST-TH total score (0–9)		2.42 ± 1.61	0	9.00
Physical subscore (0–4)		1.17 ± 1.68	0	4.00
Psychological subscore		1.24 ± 1.11	0	5.00
RMDQ (0–24)		2.44 ± 2.95	0	21.00
FABQ total score (0–66)		32.66 ± 11.85	1.00	57.00
FABQ-PA (0–24)		13.31 ± 4.69	0	24.00
FABQ-W (0–42)		19.35 ± 8.48	0	34.00
PCS total score (0–52)		11.21 ± 8.74	0	40.00
Rumination		4.63 ± 3.69	0	15.00
Magnification		2.90 ± 2.54	0	12.00
Helplessness		3.69 ± 3.59	0	18.00
HADS total score (0–42)		12.65 ± 4.95	4.00	35.00
Anxiety (0–21)		8.29 ± 2.73	1.00	19.00
Depression (0–21)		4.36 ± 2.97	0	16.00
EQ-5D-5L index (0–1)		0.91 ± 0.10	0.18	1.00
EQ-VAS (0–100)		76.81 ± 14.79	25.00	100.00

*SD* Standard deviation, *VAS* Visual analogue scale, *SBST-TH* STarT back screening tool-Thai version, *RMDQ* Roland-Morris disability questionnaire, *FABQ* Fear-avoidance beliefs questionnaire, *FABQ-PA* Fear-avoidance beliefs questionnaire about physical activity, *FABQ-W* Fear-avoidance beliefs questionnaire about work, *PCS* Pain catastrophising scale, *HADS* Hospital anxiety and depression scale, *EQ-5D-5L* EuroQol five-dimensional five-level questionnaire, *EQ-VAS* EuroQol-visual analogue scale

### Content validity

All participants completed all questionnaires with no missing data. No significant floor and ceiling effects were found in the SBST-TH (floor: ceiling = 6.5%: 0.5%).

### Factor analysis

The results of factor analyses are presented in Table 2. Two factors with an eigenvalue ≥1 (39.38% of the total variance) were extracted. The eigenvalues of two factors (psychological and physical components) were 2.45 and 1.10, respectively. Bartlett’s test of sphericity was 193.19 (*p* <  0.001) and the KMO was 0.70, demonstrating a good factor analysis of this study.

**Table 2:** Varimax-rotated factor-loading matrix of the SBST-TH

Items	Components	
	Factor 1: Psychological	Factor 2: Physical
1		0.69
2		
3	0.41	0.62^a^
4	0.67	
5		0.42
6	0.51	
7	0.47	0.48^a^
8	0.80	
9		0.62
Total variance explained	27.21	12.17

Factors loading ≥ 0.4 were presented, ^a^factor upon which item loaded most heavily

*SBST-TH* STarT back screening tool-Thai version

### Internal consistency

The Cronbach’s α values of the SBST-TH total score, physical and psychological subscores were 0.86, 0.92 and 0.76, respectively. These represent satisfactory internal consistency.

### Test-retest reliability

The mean, standard deviation and ICC for the two testings are presented in Table 3. The ICC of the SBST-TH total (ICC = 0.73, 95% confidence interval [CI] = 0.50–0.86, *p* <  0.001) and psychological subscore (ICC = 0.79, 95% CI = 0.60–0.89, *p* <  0.001) were found to have acceptable reliability.

**Table 3:** Test-retest reliability of the SBST-TH (*n* = 30)

	Mean ± SD		ICC (95% CI)	***p***-value
	First test	Second test		
SBST-TH total	2.37 ± 1.27	2.13 ± 1.28	0.73 (0.50–0.86)	< 0.001*
**SBST-TH subscores**				
Physical component	1.10 ± 0.55	1.03 ± 0.49	0.38 (0.02–0.65)	0.02*
Psychological component	1.27 ± 1.02	1.10 ± 1.00	0.79 (0.60–0.89)	< 0.001*

*SD* Standard deviation, *ICC* Intraclass correlation coefficient, *CI* Confidence interval, *SBST-TH* STarT back screening tool-Thai version

* *p* <  0.05

### Agreement

The SEMs of the SBST-TH and its psychological subscore were 0.74 and 0.58, respectively. The resultant MDCs were 2.04 and 1.60, respectively.

### Convergent validity

The correlations between the SBST-TH (including psychological subscore) and VAS (pain intensity), RMDQ, FABQ, PCS, HADS and EQ-5D-5L are presented in Table 4. The SBST-TH total score moderately to strongly correlated to other questionnaires ranging from 0.32 to 0.56. The moderation correlations were also found between its psychological subscore and other questionnaires, except the FABQ-W (*rho* = 0.29, 95%CI = 0.15–0.41), which demonstrated weak correlations.

**Table 4:** Convergent validity

Questionnaires	SBST-TH total score		SBST-TH psychological subscore	
	Spearman rank correlation (95% confidence interval)	***p***-value	Spearman rank correlation (95% confidence interval)	***p***-value
VAS pain intensity	0.45 (0.30, 0.57)	< 0.001*	0.39 (0.26, 0.50)	< 0.001*
RMDQ	0.56 (0.43, 0.69)	< 0.001*	0.49 (0.36, 0.60)	< 0.001*
FABQ total	0.42 (0.29, 0.53)	< 0.001*	0.33 (0.21, 0.43)	< 0.001*
FABQ-PA	0.35 (0.23, 0.45)	< 0.001*	0.32 (0.20, 0.44)	< 0.001*
FABQ-W	0.38 (0.26, 0.51)	< 0.001*	0.29 (0.15, 0.41)	< 0.001*
PCS total	0.41 (0.31, 0.55)	< 0.001*	0.39 (0.26, 0.51)	< 0.001*
PSC rumination	0.37 (0.24, 0.48)	< 0.001*	0.35 (0.21, 0.48)	< 0.001*
PSC helplessness	0.37 (0.23, 0.50)	< 0.001*	0.34 (0.21, 0.47)	< 0.001*
PSC Magnification	0.38 (0.25, 0.50)	< 0.001*	0.35 (0.21, 0.48)	< 0.001*
HADS total	0.38 (0.25, 0.50)	< 0.001*	0.35 (0.22, 0.45)	< 0.001*
HADS anxiety	0.34 (0.22, 0.45)	< 0.001*	0.32 (0.19, 0.44)	< 0.001*
HADS depression	0.32 (0.18, 0.44)	< 0.001*	0.30 (0.15, 0.43)	< 0.001*
EQ-5D-5L	−0.52 (−0.61, −0.40)	< 0.001*	−0.49 (−0.60, −0.38)	< 0.001*
EQ-5D VAS	− 0.41 (− 0.51, − 0.30)	< 0.001*	−0.42 (− 0.54, − 0.29)	< 0.001*

*SBST-TH* STarT back screening tool-Thai version, *VAS* Visual analogue scale, *RMDQ* Roland-Morris disability questionnaire, *FABQ* Fear-avoidance beliefs questionnaire, *FABQ-PA* Fear-avoidance beliefs questionnaire about physical activity, *FABQ-W* Fear-avoidance beliefs questionnaire about work, *PCS* Pain catastrophising scale, *HADS* Hospital anxiety and depression scale, *EQ-5D-5L* EuroQol five-dimensional five-level questionnaire, *EQ-VAS* EuroQol-visual analogue scale

* *p* < 0.05

### Discriminative validity

The AUC of the SBST-TH are presented in Table 5. The range of the SBST-TH total score was from 0.67 (95%CI = 0.55–0.78) to 0.85 (95%CI = 0.72–0.97). For the psychological subscore, the AUC range from 0.66 (0.55, 0.77) to 0.75 (0.52, 0.92). Only the AUC between the SBST-TH total score and disability (RMDQ) demonstrated excellent discrimination. Other AUC can be considered as acceptable discrimination.

**Table 5:** Discriminative validity

Reference standards	Case definition	AUCs of SBST-TH total score (95% confidence interval)	AUCs of SBST-TH psychological subscore (95% confidence interval)
Disability	RMDQ ≥7	0.85 (0.72, 0.97)	0.75 (0.52, 0.92)
Fear-avoidance beliefs	FABQ ≥43	0.72 (0.62, 0.81)	0.69 (0.60, 0.79)
Catastrophising	PCS ≥ 20	0.70 (0.58, 0.81)	0.70 (0.58, 0.81)
Depression and anxiety	HADS ≥8	0.67 (0.55, 0.78)	0.66 (0.55, 0.77)

*AUCs* Area under the curves, *SBST-TH* STarT back screening tool-Thai version, *RMDQ* Roland-Morris disability questionnaire, *FABQ* Fear-avoidance beliefs questionnaire, *PCS* Pain catastrophising scale, *HADS* Hospital anxiety and depression scale

## Discussion

The aims of this study were to translate and cross-culturally adapt the original English version of the SBST into Thai and test its psychometric properties. The findings suggest that the SBST-TH had satisfactory reliability and validity in Thai patients with non-specific LBP. The SBST-TH may be easy to understand and use in the Thai population owing to no missing data and no difficulty of collecting data in this study. The findings that no significant floor neither ceiling effects were demonstrated for the SBST-TH suggest a good content validity. Furthermore, the content validity was also verified by the expert committee. Our results were in harmony with the original version [11] and some adapted versions such as the Chinese [42] and Dutch [5].

Factor analysis identified two factors supporting the original version [11] and Persian version (43% of the variance demonstrated of the psychosocial component [factor 1] and physical component [factor 2] was 19%) [35] based on the eigenvalue. The two factors for the SBST-TH explained for 27.20% of the variance represented psychological component (factor 1) and the physical component (factor 2) was 12.17%. Another factor can be added when only the eigenvalue was considered for factor analysis (eigenvalue = 1.022). The factor can be named “central sensitisation component” (only item 2 and 11.35% of the variance). Central sensitisation, an increased responsiveness of nociceptors in the central nervous system, is an important prognosis in transitioning to chronicity [44].

High internal consistency of the SBST-TH total score (Cronbach’s α = 0.86), physical (Cronbach α = 0.92) and psychosocial subscores (Cronbach’s α = 0.76) was found, indicating a homogenous concept of the questionnaire [29]. This was similar to the total score and psychosocial subscore of the original (total score = 0.79, psychosocial subscore = 0.74) [11], Iranian (total score = 0.82, psychosocial subscore = 0.79) [45] and Persian (total score = 0.83, psychosocial subscore = 0.81) [35] versions. Unfortunately, previous studies did not provide the internal consistency of the physical subscore. The higher Cronbach’s α value of this study may come from the precise and rigorous methodologies using the standardised guidelines of cross-cultural adaptation [12]. Moreover, the ICCs of the SBST-TH total score and its psychosocial subscores were 0.73 and 0.79, respectively, demonstrating acceptable reliability supporting the original version (0.73 for total score and 0.69 for psychosocial subscore) [11] and the Brazilian version (0.79 for total score and 0.76 for psychological subscores) [46]. However, the SBST-TH demonstrated higher ICCs than some previous studies (e.g., Dutch and German versions) [5, 37]. The variation of the time interval in different studies may be a factor of the ICC variation. For the previous studies, the average time intervals were 6 days and 10 days, respectively. However, the common intervals were 2 days because a long-time interval may contribute to the patient’s clinical status.

The agreement of this study was evaluated by SEM and MDC. The SEM (an error of measurement) of the SBST-TH was 0.74 for the total score and 0.58 for the psychosocial subscore. To the best of our knowledge, only one study (Brazilian version) calculated an SEM higher than in this study (1.90 for the total score) [46]. The small SEMs led to the small MDCs, which were 2.04 and 1.60 for the total score and psychosocial subscore, respectively. These mean change scores equal to or higher than 2.04 for the total score and 1.60 for the psychosocial subscore represent a real change. Unfortunately, the MDC was not calculated from previous studies.

The correlations between the SBST-TH and all reference standard questionnaires to evaluate convergent validity were significant (Table 4). The Spearman’s correlation coefficients demonstrated that the overall SBST-TH and its psychosocial subscore moderately to strongly correlated to other questionnaires. The exceptions were the correlations between the psychosocial subscore and FABQ-W, which was weak. Remarkably, the total score was positively strongly correlated with the RMDQ (*rho* = 0.56, 95%CI = 0.43, 0.69), similar to the Japanese [8], German [37], Persian [35], and French [47] versions and negatively strongly correlated with the EQ-5D-5L (*rho* = − 0.52, 95%CI = − 0.61, − 0.40), similar to the Japanese version [8]. These findings suggest a good relationship between the SBST-TH and disability and QoL.

The discriminative validity was demonstrated by AUC for the total score and psychosocial subscore against the case definition by the reference standard questionnaires (Table 5). The highest AUC (0.85, 95%CI = 0.72, 0.97) was observed between the total score of the SBST-TH and disability (RMDQ), reflecting excellent discrimination. This represented that the total score of the SBST-TH can substantially separate cases from physical reference standard. The acceptable discrimination was found in all other AUCs. Interestingly, the AUC of the SBST-TH total score and its psychosocial subscore for the cases defined by psychosocial references (fear-avoidance beliefs, catastrophising and depression and anxiety) were found close to each other. These findings are supported by the original [11] and Japanese [8] versions.

Owing to the results from the convergent and discriminative validity, ≥ 75 of the a priori hypotheses were confirmed, leading to supporting construct validity of the SBST-TH [29]. However, this study has some limitations. First, the sample size was limited for the factor analysis considering that at least 300 participants would be required for strong factor analysis [48]. Second, the study did not have a question about referred leg pain. This led to a lack of analysis in the case defined by another physical reference for discriminative validity. Third, the sample size of test-retest reliability (*n* = 30) is doubtful and ≥ 50 participants are recommended according to the COSMIN checklist [49]. Finally, the responsiveness of the SBST-TH was not investigated.

## Conclusion

The translation and adaptation of the Thai version of the SBST were successful. Satisfactory internal consistency, reliability and construct validity of the SBST-TH were demonstrated in this study. Consequentially, the SBST-TH can be applicable in research and clinical settings to classify Thai-speaking clients with non-specific LBP into low, moderate and high risks for chronicity.

## Data Availability

The datasets used and/or analysed during the current study available from the corresponding author on reasonable request.

## Abbreviations

AUCs: Area Under the Curves
CI: Confidence Interval
EQ-5D-5L: The EuroQol Five-Dimensional Five-Level questionnaire
EQ-VAS: EuroQol-Visual Analogue Scale
FABQ: Fear-Avoidance Beliefs Questionnaire
FABQ-PA: Fear-Avoidance Beliefs Questionnaire about Physical Activity
FABQ-W: Fear-Avoidance Beliefs Questionnaire about Work
HADS: Hospital Anxiety and Depression Scale
ICC: Intraclass Correlation Coefficient
LBP: Low Back Pain
MDC: Minimal Detectable Change
PCS: Pain Catastrophising Scale
QoL: Quality of Life
RMDQ: Roland-Morris Disability Questionnaire
SBST: STarT Back Screening Tool
SBST-TH: STarT Back Screening Tool - Thai version
SD: Standard Deviation
SEM: Standard Error of Measurement
VAS: Visual Analogue Scale
